# Ultrastructural and proteomic evidence for the presence of a putative nucleolus in an Archaeon

**DOI:** 10.3389/fmicb.2023.1075071

**Published:** 2023-02-02

**Authors:** Parsifal F. Islas-Morales, Anny Cárdenas, María J. Mosqueira, Luis Felipe Jiménez-García, Christian R. Voolstra

**Affiliations:** ^1^Programa de Doctorado en Ciencias Biomédicas, Facultad de Medicina, UNAM, Mexico City, Mexico; ^2^UNESCO Chair on Science Diplomacy and Scientific Heritage, Instituto de Biología, UNAM, Mexico City, Mexico; ^3^Red Sea Research Center (RSRC), Biological, Environmental Sciences, and Engineering Division (BESE), King Abdullah University of Science and Technology (KAUST), Thuwal, Saudi Arabia; ^4^Department of Biology, University of Konstanz, Konstanz, Germany; ^5^Department of Biology, American University, Washington, DC, United States; ^6^NEOM, Saudi Arabia; ^7^Department of Cell Biology, Faculty of Sciences, UNAM, Mexico City, Mexico

**Keywords:** AgNOR, Archaea, nucleolus, evolution, proteomics, *Saccharolobus*, TACK, microscopy

## Abstract

Nucleoli are subcellular compartments where transcription and maturation of pre-ribosomal RNAs occur. While the transcription of ribosomal RNAs is common to all living cells, the presence and ultrastructure of nucleoli has been only documented in eukaryotes. Asgard-Archaea, the closest prokaryotic relatives of eukaryotes, and their near relatives TACK-Archaea have homologs of nucleolar proteins and RNAs in their genome, but the cellular organization of both is largely unexplored. Here we provide ultrastructural and molecular evidence for the presence of putative nucleolus-like subcellular domains in the TACK crenarchaeon *Saccharolobus solfataricus* (formerly known as *Sulfolobus solfataricus*). Transmission electron microscopy (TEM) revealed consistent electron-dense fibro-granular compartments, also positive to the specific silver staining for nucleolar organizer regions (AgNOR). TEM also confirmed that ribosomal DNA (rDNA) is spatially distributed in non-random, clustered arrays underlying fine structures, as observed by ultrastructural *in situ* hybridization (UISH). To further explore these observations, proteomic sequencing of isolated bands from AgNOR-stained protein gels was conducted and compared against a compiled inventory of putative nucleolar homologs from the *S. solfataricus* P1 genome. Sequenced AgNOR-sensitive peptides encoded homologs of eukaryotic nucleoli proteins, enriched for nucleolus-related functions. Our results provide first evidence that subcellular domains of nucleolar-like nature are not exclusive to eukaryotes. Based on our data, we propose a model for a putative nucleolus in *S. solfataricus*. Whereas technical limitations and further aspects remain a matter for future functional studies, our data supports the origin of nucleoli within the common ancestor of Eukarya and TACK-Archaea, based on a two-domain tree of life.

## 1. Introduction

Nucleoli are fibro-granular subcellular domains that are the site of ribosomal gene expression, maturation, and regulation. Ribosome biosynthesis comprises more than 50% of eukaryotic gene expression and dictates the formation of nucleoli (Hernandez-Verdun, 2006; Pederson, 2011). The disruption and formation of nucleoli (nucleologenesis) in eukaryotes occurs during mitosis, particularly in telophase, when pre-nucleolar bodies (PNBs) are formed and then recruited as soon as rDNA transcription re-initiates at chromosomal domains called Nucleolar Organizer Regions (NORs). When the synthesis of pre-rRNA starts, a visible aggregation of ribonucleoproteins forms the nucleolus (Jiménez-García et al., 1989, 1994; Dundr et al., 2000; Hernandez-Verdun, 2006). Mature nucleoli are conspicuous, dynamic, and multi-functional compartments. They are made of rDNA, diverse types of RNAs, and over 300 proteins involved in the fine-tuning of ribosome biosynthesis that are coordinated with processes such as programmed cell death, metabolic regulation, cell differentiation, stress, and aging (Andersen et al., 2005; Grummt, 2013). Given this compositional complexity, the identification and study of nucleoli in most species have initially relied on ultrastructural features and cytochemistry, with the first choice of diagnostic being the presence of an electron-dense fibro-granular domain at the ultrastructural level that is positive to highly specific ammoniacal silver staining of the nucleolar organizer regions (AgNOR) (Smetana, 2011). Although a high degree of morphological heterogeneity is present across nucleoli of different lineages, those diagnostics have remained useful and have become nucleolar characters across phylogeny (Heath, 1980; Thiry and Lafontaine, 2005). Thus, our main understanding of nucleolus molecular physiology comes from functional studies developed in a small group of model organisms where specific antibodies and cell cycle markers are available (mammalian, amphibian, yeast, and plants), leaving a vast field of new research to be unexplored across many lineages and species (Islas-Morales et al., 2021).

In line with this, although ribosomal gene expression and maturation are common to all living cells, the presence of nucleoli has been attributed exclusively to eukaryotic cells, so far. Despite this paradigmatic view, some eukaryotic nucleoli remained elusive over decades. For instance, the ultra-small nucleolus in the protozoan *Giardia lamblia* was not evidenced until 2008 following classical ultrastructural observation and complimentary molecular approaches (Jiménez-García et al., 2008). The lack of a nucleolus in *G. lambia* was at the heart of the debate over the origin of eukaryotes, i.e., the idea of diplomonads and amoebas as proto-eukaryotes, as posited in the archeozoa hypothesis (Cavalier-Smith, 2002). Based on the ultrastructural evidence of a nucleolus in early branching eukaryotes, it is now accepted that the nucleolus existed in the Last Common Ancestor of Eukaryotes (LECA) (Jiménez-García et al., 2008).

The Tree of Life (ToL) has changed exhaustively over time and determining where and when the nucleolus originated could become a new field of study in evolutionary cell biology (Islas-Morales et al., 2021). In a two-domain ToL, where Eukarya and Archaea form a single life domain (Gribaldo et al., 2010), the nucleolus may have evolved gradually among and within sister lineages, rather than exclusively within the Eukarya (from the LECA). For instance, the recently highlighted Asgard-Archaea are the closest relatives of eukaryotes, including many unculturable and newly discovered groups of archaea such as the so-called Lokiarchaea that may display eukaryotic cell features (Spang et al., 2015; Liu et al., 2021). Also, within the Asgard-Archaea closest culturable relatives, the TACK Archaea, a significant number of eukaryotic homologies have been found *in silico* (Guy and Ettema, 2011). In line with this, homologs of major nucleolar elements such as fibrillarin and small nucleolar RNAs have been reported in TACK Archaea (Omer et al., 2000). Nucleolar homologs, for example, have been studied *in silico* in *S. solfataricus*, a culturable TACK-archaeon. Further, when expressed in transfected frog cells, some small nucleolar RNAs from *S. solfataricus* were observed to accumulate in the (eukaryotic) nucleolus (Omer et al., 2000). With the availability of the nucleolar proteomes of yeast and human (Andersen et al., 2005), studies now support that most nucleolar homologs across prokaryotic lineages are present mainly in Archaea (Staub et al., 2004). Furthermore, clusters of homologous genes for ribonucleoprotein complexes such as the SSU processome are also present in Archaea, but not in Bacteria (Feng et al., 2013). However, previous studies have proposed that a nucleolus-like compartmentalization could also occur for the transcription in the bacterium *Escherichia coli*, based on the co-localization of DNA polymerase and the bacterial nucleolar homolog NusB (Jin et al., 2017; Mata Martin et al., 2018). Yet, the search for a putative nucleolus ultrastructure in Archaea and Bacteria using experimental and ultrastructural approaches remains unexplored in the extant literature. We posit that much can be discovered regarding the understanding of archaeal cell structure and evolution when employing omics-oriented microscopy at the nano-level (Islas-Morales et al., 2021). Motivated by the above, we searched for initial evidence of a putative nucleolus ultrastructure in the crenarchaeon *S. solfataricus*. We chose *S. solfataricus* because it is a culturable organism from the TACK Archaea superphylum. Our approach followed ultrastructural examination with subsequent exploration and integration of molecular data. Although our data support the presence of a nucleolus in an Archaeon, technical limitations and further aspects remain to be addressed in future studies.

## 2. Materials and methods

### 2.1. *Saccharolobus solfataricus* cultivation

Cultivation was done according to standard procedures (Robertson, 2007). Briefly, the type strain *S. solfataricus* P1 DMSZ 1616 from the German Collection of Microorganisms and Cell Cultures^1^ was grown at 80°C in media DSMZ88^2^ and DSMZ182.^3^ From a two liters bioreactor, samples were taken for processing in Transmission Electron Microscopy (TEM). Cells were harvested at log phase *S. solfataricus* with a cell density of > 10^9^ cells/mL measured by optical density in a spectrophotometer at 600 nm.

### 2.2. Microscopy

We examined *S. solfataricus* using Light and Transmission Electron Microscopy (TEM). In the absence of specific antibodies, we focused on nucleolar cytochemistry, morphology, and cytogenetics, such as AgNOR staining positivity, fibro-granular nanodomains, and rDNA clustering (Goodpasture and Bloom, 1975; Scheer et al., 1993; Hernandez-Verdun, 2006; Pederson, 2011).

#### 2.2.1. Light microscopy

Observations of *S. solfataricus* cells in bright field-, phase contrast-, and dark field-microscopy were performed on an Olympus BX41 upright microscope to confirm culture viability using DAPI stain. For visual assessment of AgNOR staining using light microscopy, cells were fixed and processed in slides according to previously described protocols (Jiménez-García, 1998; Andersen et al., 2005). We repeated this technique 10 times independently, counting 3,000 cells for each replicate. Following this, the totality of cells was positive to AgNOR staining, which became evident depending on the focal plane.

#### 2.2.2. Transmission Electron Microscopy (TEM)

Standard TEM sample preparation with modifications was carried out for *S. solfataricus* cells (Robertson, 2007; Segura-Valdez et al., 2013). For general morphology with standard heavy metals contrasting, fixation was performed as follows: 300–500 mL of filtered (Millipore 5 μm) cell culture was fixed in 2.5% glutaraldehyde and 4% paraformaldehyde for 1 hour. Fixatives were added directly to the culture. Intermediate washes were carried out in a buffer composed of the salts of medium DMSZ182 adjusted to pH 4 to prevent osmotic damage. After fixation, cells were gently centrifuged at 2800 g and re-suspended in 1 mL of filtered 1% agarose. The suspension was poured into a small Petri dish reaching 2 mm height. This cell mat solidified and was prevented from drying using DMSZ88 medium-buffer. The mat was cut into 2 mm^2^ pieces, each was dehydrated with ethanol: 30%; 50%; 70%; 80%; 90%; 95%; 3 × 100% at 10-min intervals at low temperature. Pre-inclusion was carried out with three washes in 100% propylene oxide at 5-min intervals and a final mixture of propylene oxide and Epon 611 (1:1 v/v) for 48 h, and subsequent polymerization in pure resin at 60°C. General contrast staining was performed using UranyLess EMS stain (Electron Microscopy Sciences #22409) and lead citrate under CO_2_ limited conditions (Robertson, 2007). Imaging was done using a JEOL 1010 Transmission Electron Microscope at 80 kW.

#### 2.2.3. Ultrastructural AgNOR silver impregnation

This specific staining technique for Nucleolar Organizer Regions (NOR) was performed in *S. solfataricus* cells according to previously described protocols for single cells providing superior results (Jiménez-García, 1998; Jiménez-García et al., 2008). Briefly, cells were double-fixed in 2.5% glutaraldehyde for 1 hour and then washed in Carnoy’s solution (glacial acetic acid and 75% ethanol at 1:3 v/v) for 5 min. Cells were rehydrated in a series of 70% and 50% ethanol and bi-distilled water at 10-min intervals. Cells were embedded in 1% agarose cubes as described in section 3 to prevent loss of sample. The AgNOR reaction was started by adding drops of 50% silver nitrate solution (prepared with 1 g AgNO_3_ in 2 mL distilled water) and incubating for 10 min in hot water (70°C), then washing 5 times with ice-cold water, and then adding 4 drops of the NH_4_ reagent solution (4 g AgNO_3_; 5 mL double-distilled water; 5 mL of concentrated NH_4_OH at pH 12–13) and four drops of catalyst. The catalyst used consisted of a 3% formaldehyde solution that has been neutralized with sodium acetate crystals and adjusted to pH 5–6 with formic acid. After the cubes displayed a yellowish coloration, they were washed five times in ice-cold water. Subsequently, we proceeded with standard EM dehydration and embedding in Epon 600, as described under the TEM section. We repeated this procedure three times independently, sampling 30 random cells in each replicate. We calculated an AgNOR average signal density of 80% from a mean value of *n* = 24 ± 2 cells showing a positive signal. Of note, some positive cells may have escaped our observation due to the focal plane.

#### 2.2.4. Ultrastructural *in situ* hybridization of 16S and 23S ribosomal regions

We performed ultrastructural *in situ* hybridization (UISH) of 16S and 23S rDNA genes, because nucleoli are ribonucleoprotein compartments. According to standard procedures (Segura-Valdez et al., 2013), fixation was performed as follows: 300–500 mL of filtered (Millipore 5 μm) cell culture was fixed in a glutaraldehyde 2.5% and paraformaldehyde 0.5% mixture for 1 hour. Intermediate washes and dehydration were performed as detailed under general morphology. Embedding was carried out after dehydration using an increasing proportion of Lowicryl K4M and Ethanol (1:2; 1:1; 2:1; v/v) and absolute resin for 24 h at −20°C. K4M polymerization was carried out at low temperature under a UV light source. Imaging was done using a JEOL 1010 Transmission Electron Microscope operating at 80 kW.

To prepare the probes, aliquots of 1.5 mL of archaeal cultures were centrifuged at 10,000 g for 10 min, cell pellets were washed twice with 200 μL of sterile water and resuspended in 100 μL of ATL lysis buffer (Qiagen) to proceed with the DNA extraction using the DNeasy Blood & Tissue Kit (Qiagen). UISH was performed according to standard protocols (Segura-Valdez et al., 2013). Briefly, primers 27F and 1492R (Lane, 1991) and 189F and 2490R (Hunt et al., 2006) were used to amplify 16S and 23S rRNA gene regions from a *S. solfataricus* cell culture, respectively. Amplification was conducted until reaching a rDNA amplicon concentration of 1 mg/mL. UISH probes were generated using a nick translation mix following standard protocols (Segura-Valdez et al., 2013). The labeling reaction with Biotin dUTP was performed on ice with 16 μL sterile double distilled water containing 1 μg rDNA with equimolar amounts of the generated 16S and 23S amplicons and 4 μL of Biotin-Nick Translation Mix (Sigma Aldrich). The nick translation mix contained: 5 × concentrated reaction buffer, 50% glycerol, DNA Polymerase 1, DNase 1, 0.25 mM each of dATP, dCTP, dGTP, 0.17 mM dTTP, and 0.08 mM Biotin-dUTP. After brief centrifugation, the mixture was incubated at 15°C for 90 min and chilled to 0°C. The reaction was stopped with 1 μL 0.5 M EDTA (pH 8.0) and heating at 65°C for 10 min. The length of the probes (200–500 nucleotides) was checked using an agarose gel with a DNA size marker. Probes were stored in TE buffer (10 mM Tris HCl, 1 mM EDTA, pH 8.0) at −20°C. The hybridization reaction was performed “post-embedding” on 200 Mesh gold grids carrying sections from *S. solfataricus* in Lowicryl K4M resin. In order to hybridize, the native DNA present in the TEM sections on the gold grids as well as the amplicon DNA present in the hybridization mix (composed of 12 μL of probe amounting to ~170 ng/μL, 2.5 μL of 20x SSC, and 12.5 μL of formamide), both were denatured separately at 100°C in bowling water for 5 min. Hybridization took place by placing one drop of hybridization mix on the grid with the TEM sections and incubating at 37°C overnight, preventing evaporation. Subsequently, an immunoreaction was performed using GOAT-ANTI-BIOTIN antibody coupled to 10 nm Nanogold (EMS). This was incubated in PBS (1:10) for 30 min. After washing repeatedly in PBS and water, grids were contrasted with UranyLess stain (Electron Microscopy Sciences #22409) and observed in a Transmission Electron Microscope FEI Tecnai twin at 80 kW. We repeated this procedure three times independently, sampling 30 random cells in each replicate. We calculated an average hybridization signal density of 82% from a mean value *n* = 24.6 ± 1.2 cells exhibiting a positive signal. Of note, some positive cells may have escaped our observation due to the focal plane.

### 2.3. Proteomics

For our proteomics approach, we employed an *in vitro* variant of the AgNOR method by staining *S. solfataricus* protein extracts that were separated on an SDS-PAGE protein gel (Lischwe et al., 1979). Our strategy was to utilize the conditions and specificity of the AgNOR method employed for TEM. This resulted in a discrete banding pattern with remarkable contrast in the SDS-PAGE protein gel (Supplementary Figure S4) in contrast to CBB staining (Supplementary Figure S5). We excised AgNOR-positive bands that contained AgNOR-labeled peptides and subjected them to ancillary mass spectrometry analysis coupled to MALDI-TOF sequencing (Chevallet et al., 2006). The detailed procedures were carried out as detailed below.

#### 2.3.1. AgNOR staining in SDS-PAGE gels from *Saccharolobus solfataricus* protein extracts

AgNOR staining in SDS-PAGE gels was done according to established procedures (Lischwe et al., 1979; Buys and Osinga, 1984; Trerè, 2000). Briefly, 300–500 mL of filtered (Millipore 5 μm) cell culture was centrifuged and concentrated into a pellet. The resuspended pellet was denatured at 100°C for 3 min in Laemmli buffer adding 4x SB containing 400 mM DTT (31 mg/500 μL) to a final concentration of 1×. This solution was then incubated at 90°C for 2 min and loaded onto a 10% 1-D polyacrylamide gel and run at ~10–12 mA. The SDS-PAGE gel was fixed in Carnoy’s solution for 1 hour, washed with deionized water, and incubated for 2 hours in borate buffer (0.1 M Na_2_S0_4_ and 0.005 M Na_2_B_4_0_7_, pH 9.2). Incubation with 50% aqueous silver nitrate took place overnight at 50°C. Bands were already apparent after this step. The gel was placed in 3% formalin to contrast it more clearly (Goodpasture and Bloom, 1975; Lischwe et al., 1979). This method resembles the condition for nucleolar staining in cytological preparations (Trerè, 2000).

#### 2.3.2. Mass spectrometry preparation and analysis

In preparation for MALDI-TOF, we followed an ancillary silver distaining protocol for mass spectrometry and subsequent standard procedures for in gel digestion and peptide extraction (Gharahdaghi et al., 1999; Chevallet et al., 2006; Shevchenko et al., 2006). Briefly, gel bands were covered in 30 mM aqueous potassium ferricyanide C_6_N_6_FeK_3_ and 100 mM sodium thiosulfate Na_2_S_2_O_3_ in equal volumes until the stain was removed approximately 6 min after washing with water. The pieces were soaked in 200 mM ammonium hydrogen carbonate (NH_4_)HCO_3_ for 20 min (approximately 0.15 mL per gel slice), washed again, and stored dry at −20°C. In-gel protein digestion was performed as follows: to extract the protein part for MALDI-TOF, the distained gel sections were reduced in 10 mM of dithiothreitol (DTT) in 100 mM (NH_4_)HCO_3_ at 37°C for 30 min, and then alkylated in 50 mM of iodoacetamide (IOA) in 100 mM (NH_4_)HCO_3_ for 1 hour, and subsequently washed, dehydrated, and rehydrated. Finally, the gel slices were digested with Trypsin (12.5 ng/μL) at 37°C for 16 h. Proteins were then extracted with 5% acetic acid and 50% acetonitrile (ACN, CH₃CN). After extraction, peptides were washed twice in 0.1% trifluoracetic acid TFA (*CF*₃COOH), eluted in 75% ACN, and completely dried by SpeedVac. The lyophilized protein extract was re-dissolved with 0.1% FA in HPLC-grade H_2_0 and quantified by NanoDrop at A280. Concentrations were normalized across samples for DIA/SWATH-MS analysis. Indexed retention time (iRT) standards (Biognosys, Ki30021) were added to the ready to inject peptide mixture at a 3:10 ratio (v/w) to Ultraflex III MALDI-TOF/TOF MS (Bruker).

### 2.4. Genomics

For our genomic analysis, we compiled a list of candidate nucleolar protein homologs in the *S. solfataricus* P1 genome for subsequent comparison to protein complements of eukaryotic nucleoli.

Additionally, we conducted a Gene Ontology (GO) analysis to identify enriched biological processes and molecular functions prevalent among precipitated proteins derived from the MS analysis of the AgNOR stained peptides in SDS-PAGE gels.

#### 2.4.1. Nucleolus related genes in *Saccharolobus solfataricus*

Amino acid translated genes of the genome of *S. solfataricus* strain P1 (GenBank accession number NZ_LT549890.1) were annotated using the KEGG Automatic Annotation Server (KAAS) (Moriya et al., 2007) with the BBH (bi-directional best hit) method against the representative eukaryotic set of genes. Only gene features with KEGG assignments affiliated to the pathway “Ribosome biogenesis in eukaryotes” (ko03008) were considered in the analysis. In addition, a hidden Markov model (HMM) profile search was performed using HMMER3 v.3.1b2 (Eddy, 2011) to identify protein domains of the nucleolus reported previously (Staub et al., 2004).

#### 2.4.2. Gene Ontology (GO) enrichment of AgNOR-stained proteins extracted from SDS-PAGE

Peptide sequences of the proteins extracted and analyzed with MALDI-TOF were aligned to the translated gene features of the *S. solfataricus* strain P1 genome (GenBank accession number NZ_LT549890.1) using the BLASTp algorithm (Supplementary Data). Only hits with a 100% identical match were allowed (no mismatches). All protein features were annotated against the UniProtKB/SwissProt database (The UniProt Consortium, 2018) and only hits with an E-value <10^−3^ were considered. GO annotations were obtained from UniProt IDs by parsing a GOA gene association file (available at: ftp.ebi.ac.uk/pub/databases/GO/goa/UNIPROT/). A Gene Ontology (GO) enrichment analysis was done on the precipitated proteins using the “weight01” method implemented in the R package TopGO v.2.42.0 (Alexa and Rahnenfuhrer, 2022). Resulting *p* values were adjusted for multiple testing using the false discovery rate (FDR) method (Benjamini and Hochberg, 1995), and GO categories with an adjusted value of *p* (*q*-value) below 0.05 were considered enriched.

## 3. Results

**Figure 1:**
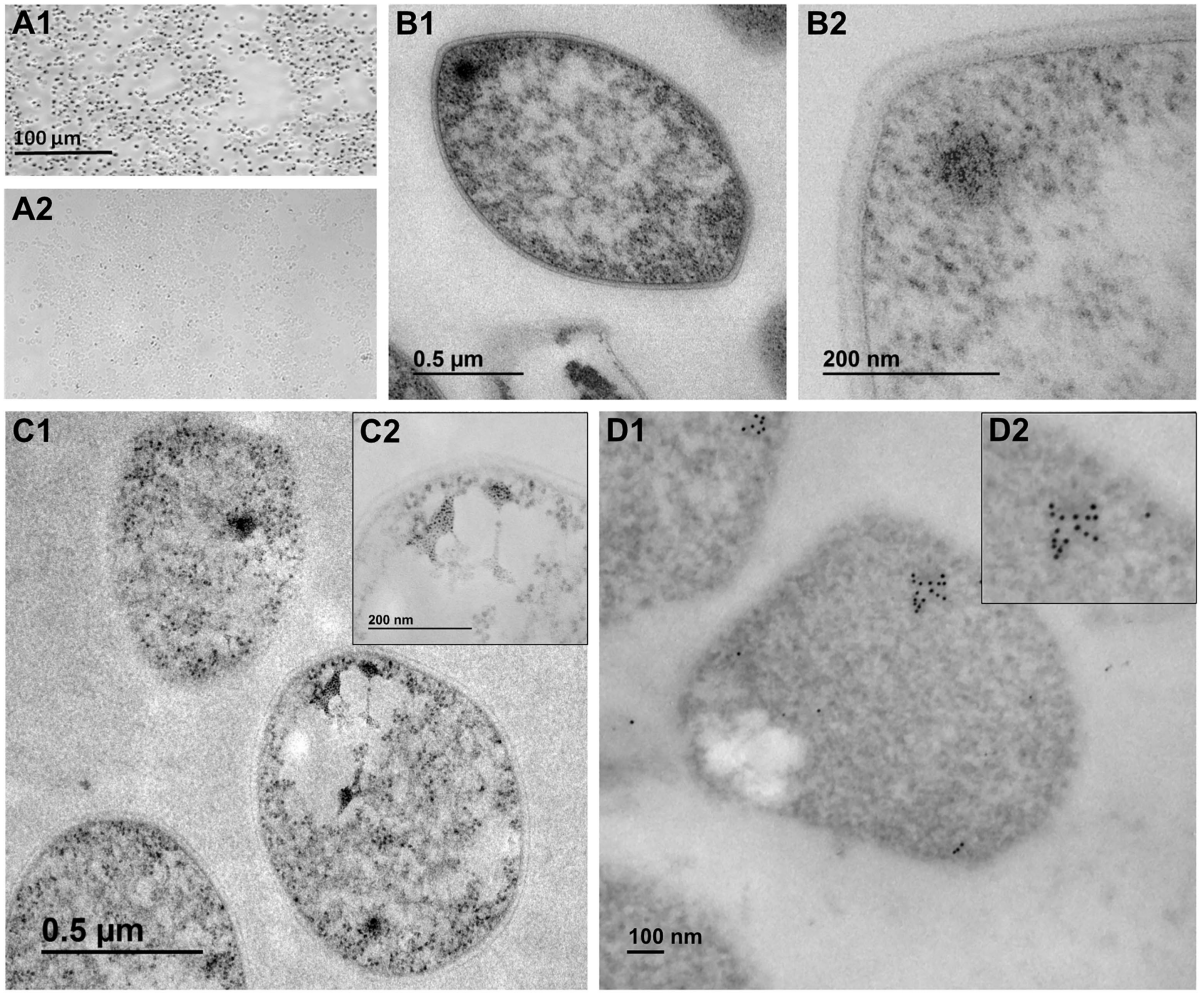
Light and electron microscopy observations support the presence of putative nucleolus-like domains in the crenarchaeon *Saccharolobus solfataricus* at the ultrastructural level. **(A1)** Light microscopy of the AgNOR reaction. **(A2)** Light microscopy of unstained *S. solfataricus* cells (negative control). **(B1)** Transmission Electron Microscopy (TEM) of *S. solfataricus* cells, with **(B2)** detail of discrete fibro-granular structures. **(C1)** Ultrastructural AgNOR impregnation of subcellular structures, with **(C2)** high magnification of AgNOR positive subcellular structures. **(D1)** Ultrastructural *In situ* hybridization (UISH) of 16S and 23S rDNA clusters, with **(D2)** magnified image showing an underlying electron-dense region.

By light microscopy, we consistently noticed a strong AgNOR impregnation in the totality of examined cells (Figures 1A1,A2). By general TEM, we frequently observed electron-dense intracellular bodies with an outstanding fibro-granular morphology in *S. solfataricus* cells at low and high magnifications (Figures 1B1,B2, 2; Supplementary Figures S1, S2), highly similar to the ultra-small nucleoli from *G. lamblia* (Jiménez-García et al., 2008). Furthermore, we observed a conspicuous, specific, and strong AgNOR silver impregnation in *S. solfataricus* cells by electron microscopy (Figures 1C1,C2). This result is relevant because ammoniacal silver impregnation of the Nucleolar Organizer Regions (AgNOR) is a specific technique for argyrophilic proteins associated to the NOR and evidences the presence of NORs, pre-nucleolar bodies, and consequently nucleoli at the ultrastructural level (Goodpasture and Bloom, 1975; Jiménez-García et al., 1994; Pederson, 2011; Grummt, 2013). Thus, for the first time we provide evidence that fibro-granular structures and AgNOR signals are present as discrete domains in Archaea.

**Figure 2:**
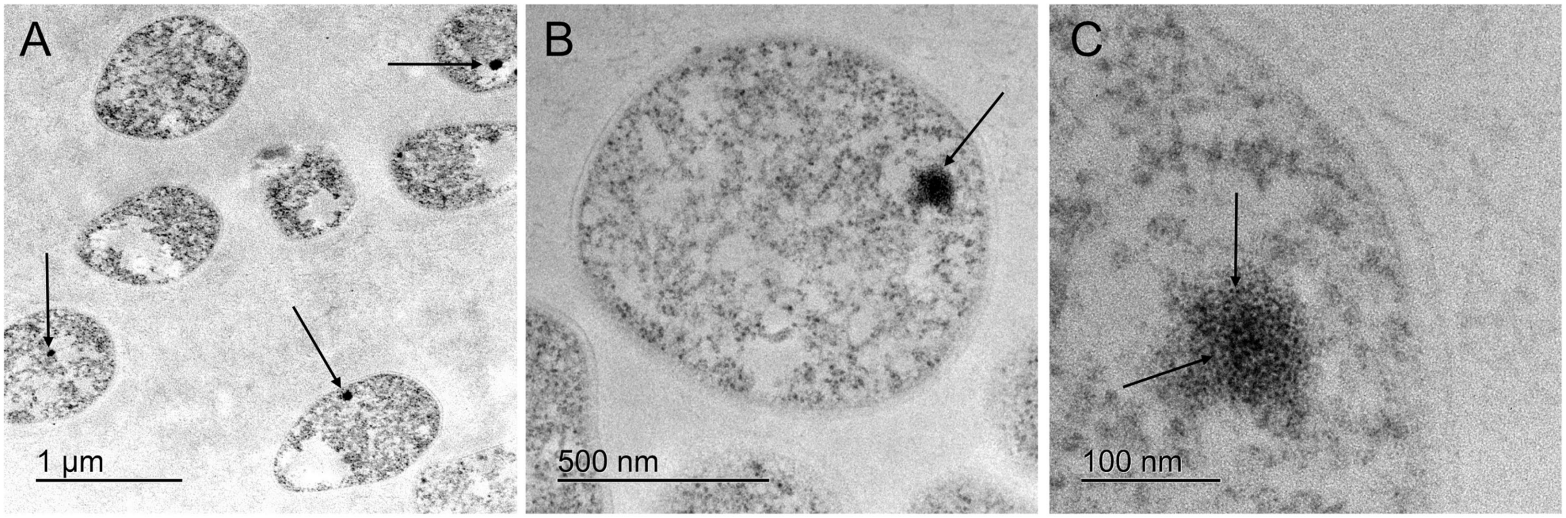
Transmission electron microscopy of fibro-granular, putative nucleolus-like domains in cells of the crenarchaeon *S. solfataricus*. **(A)** Arrows show electron dense regions, present in most cells at low magnification. **(B)** Single *S. solfataricus* cell with an electron dense domain, in which a fibro-granular morphology is evident. **(C)** Single *S. solfataricus* cell at high magnification. Arrows show the electron dense region where granules in the central part and fibers toward the outside can be observed. A differentiation of structural substrate is evident in comparison to the surrounding cytoplasm.

Ultrastructural *in situ* hybridization (UISH) of 16S and 23S rDNA under denaturing conditions showed expected localization of both rDNA and rRNA. The distribution of ribosomal genes and their transcripts in *S. solfataricus* may correspond to single and discrete subcellular clusters with an underlying electron-dense region (Figures 1D1,D2). Notably, for UISH under non-denaturing conditions (Supplementary Figure S3), it is expected to only detect a signal from rRNA. Despite broader signal distribution, a concentration of discrete subcellular clusters was evident, corresponding to the observation under UISH under denaturing conditions. Although colocalization of rDNA and rRNA cannot be claimed, observations from UISHs under denaturing and non-denaturing conditions are complementary and suggest that ribosomal transcripts may be spatially related to a subcellular domain. According to nucleologenesis, while NORs are strictly associated with rDNA, pre-nucleolar bodies (PNBs) and other discrete elements of nucleolar origin do not necessarily contain ribosomal genes (Jiménez-García et al., 1989, 1994). In this sense, our UISH observations are consistent with the notion of a single locus of rDNA in the *S. solfataricus* genome and complement the observation of multiple AgNOR silver impregnations associated to discrete subcellular domains with remarkable contrast. Following the above, we conclude that *S. solfataricus* AgNOR-sensitive proteins accumulate at specific locations and share a similar composition with eukaryotic NORs and nucleoli.

In addition to the AgNOR-based stainings, it is important to consider the consistency of ultrastructural observations using general contrast techniques without specific stains. Figure 2A shows electron-dense subcellular domains in *S. solfataricus*. Figures 2B,C display higher magnifications and detail of a nucleolar-like fibrogranular morphology of subcellular domains in *S. solfataricus*, resembling a typical nucleolar ultrastructure. Despite nucleolar morphology being highly heterogeneous across the ToL, especially in early branching protists, our observations are consistent with a nucleolus that features two main morphological components: a *pars fibrosa* and a *pars granulosa*, which constitute accepted criteria for nucleoli outside amniotes following previous studies (Thiry and Lafontaine, 2005).

To explore which molecular elements underlie the newly discovered nucleolus-like structure, we stained *S. solfataricus* protein extracts using the AgNOR method and subsequently separated them on a SDS-PAGE protein gel. AgNOR-positive bands were excised and sequenced, resulting in 1,618 distinct peptides of which 1,527 exhibited perfect matches (100% similarity) to 376 proteins in the *S. solfataricus* P1 genome, referred to in the following as “precipitated proteins” (Supplementary Tables S1, S2). These included nucleolus proteins, such as: NOP1 (fibrillarin), NOP5, L7Ae, L30Ae, L31Ae, and PUA (pseudouridine synthase), all of which are involved in ribosomal RNA (rRNA) maturation and small nucleolar RNA (snoRNA) metabolism. We also found evidence of RNA polymerase, essential to form nucleolar organizers by initiating rRNA transcription. Gene Ontology (GO) analysis corroborated that “translation”, “structural constituent of the ribosome”, “rRNA binding”, and “unfolded protein binding” were prominent processes among precipitated proteins and significantly enriched (FDR ≤ 0.05, Supplementary Table S3). This suggests that proteins in the putative nucleolus-like compartment harbor functions associated with ribosomal expression and RNA binding and are potentially capable of forming macromolecular ribonucleoproteins, which are the core components of non-membranous organelles such as nucleoli and Cajal bodies (Dundr et al., 2000; Pederson, 2011). Of note, as known from eukaryotic nucleoli, not all nucleolar proteins are argyrophilic and thus amenable to AgNOR staining (Sirri et al., 2000). Additionally, not all eukaryotic nucleolar proteins have homologs in Archaea. Consequently, some known AgNOR-positive proteins from Eukarya, such as nucleophosmin and nucleolin were not present in our proteomics analysis, as expected (Lischwe et al., 1979; Hernandez-Verdun et al., 1980).

To obtain a comprehensive view of nucleolar elements in Archaea, we used genomics analysis to complement identified proteins based on AgNOR staining. Using alignment-based approaches, we identified 18 genes related to the KEGG pathway “ribosome biogenesis” (Supplementary Table S4) and a further 41 genes (Supplementary Table S5) identified by the presence of previously reported nucleolus-related domains (Staub et al., 2004). The identified 59 gene homologs encoded for 36 distinct proteins. We propose that these 36 proteins constitute a ‘minimal set’ of nucleolar elements (Supplementary Table S6). Ten of these proteins were confirmed by AgNOR-stained proteomic sequencing (see above).

In summary, various lines of evidence confirm the presence of nucleolar elements in *S. solfataricus* both *in situ* and *in silico* (Supplementary Figure S6). First, the existence of refringent, electron-dense, and AgNOR-positive nucleolar-like domains is supported by optical and transmission electron microscopy. This observation is consistent with the genome architecture and non-random distribution of 16S/23S rDNA sequences and accumulating rRNA as evidenced by ultrastructural *in situ* hybridization (notably, UISH under denaturalizing conditions can detect rDNA and rRNA). Further, peptide sequencing of AgNOR-stained protein gel bands supports the notion that the silver-stained proteins in the putative nucleolus-like compartments are associated with nucleolus-ascribed structures and functions, such as RNA-protein interactions and pre-rRNA maturation. Further, genomics analysis concludes our understanding of nucleolar elements in *S. solfataricus* by linking silver-stained proteins with a core set of nucleolus homologs, including non-argyrophilic nucleolar proteins that escaped AgNOR-based peptide sequencing.

## 4. Discussion

**Figure 3:**
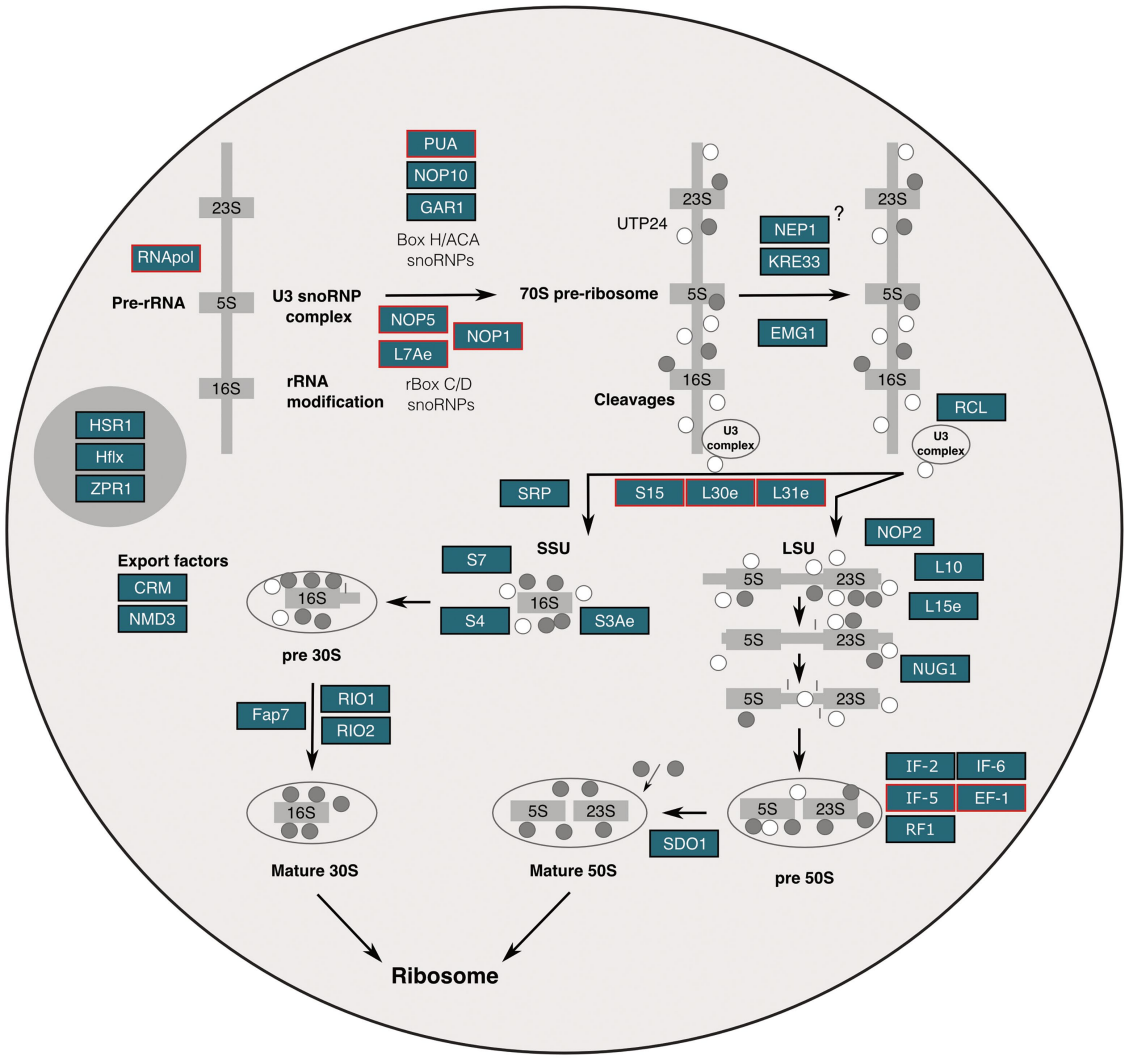
Molecular organization of the putative nucleolus of *S. solfataricus* based on ultrastructural, molecular, and genomics evidence. Nucleolar elements identified in the *S. solfataricus* genome through homology search or by means of protein domain prediction are colored in blue boxes; proteins supported by proteomic evidence from sequencing of AgNOR fractions are highlighted in red.

Our comprehensive data allowed us to conceptualize a putative ribosome biogenesis pathway in *S. solfataricus*. This is an initial and complementary proposal, which also should be analyzed in light of extensive works on the evolution of ribosome biogenesis in Archaea (Ebersberger et al., 2014; Birikmen et al., 2021). Using the KEEG pathway ko03008 (“ribosome biogenesis in eukaryotes”) as a basis and adapting it to an archaeal cell model, we hypothesize that the identified protein homologs co-reside in the putative nucleolus-like domains, which provides additional insights worthy of discussion (Figure 3). For instance, argyrophilic proteins L7Ae and fibrillarin (NOP1) as well as non-argyrophilic EMG1 and NOP2 stand out because they are part of the nucleolar organizer C/D and H/ACA box ribonucleoprotein (Pederson, 2011). This complex directs the highly dynamic post-transcriptional modification of rRNA, in the form of methylations and pseudouridylations (Hernandez-Verdun, 2006; Pederson, 2011). The study of how nucleolar elements move and organize as part of this process has been instrumental in understanding nano-scale nucleic acid-protein interactions and its relation to protein disease and aging research (Jiménez-García et al., 1994; Grummt, 2013). We think that our findings may provide an ancestral view of this kind of nano-scale dynamism that could further contribute to the study of nucleolus biology and related diseases through provision of a minimal model.

Apart from this, the phylogenetic relationship of NOP1 (fibrillarin) and NOP2 deserves further discussion. While NOP2, which codes for archeosine transglycosylase in Archaea is not argyrophilic, it shares a methyltransferase domain with the AgNOR-positive NOP1 (Rodriguez-Corona et al., 2015). Based on this, we hypothesize that during the early stages of nucleolar evolution, argyrophilic proteins may have aggregated around acidic DNA environments, which then served as a subsequent recruitment site for factors involved in post-transcriptional rRNA modification. Many proteins in the putative nucleolus share domains and may perform different functions that became more specialized over time, i.e., adapted further in species with different cellular architectures. Of note, our understanding of how proteins are organized inside the archaeal cell and where cellular processes occur spatially is still limited. For this reason, it caught our attention that protein domains of the signal recognition particle protein SRP19 were present in our data (Supplementary Table S6). In eukaryotes, SRPs are associated with the nucleoli in eukaryotes because SRP-dependent mRNA targeting for proteins destined to enter the secretory pathway via the endoplasmic reticulum (ER) and subsequent packaging into vesicles in the Golgi apparatus occurs in the nucleolus (Pederson, 2011). However, since Archaea lack an ER and Golgi apparatus, we hypothesize that SRPs are involved in other, potentially similar functions, given their spatial relationship to the nucleolus, which would then precede their secretory signaling functioning, as suggested by our data.

Notably, in contrast to eukaryotic translation where the nucleolus is separated by a nuclear envelope, *S. solfataricus* rRNA transcription and translation occur simultaneously and can be linked spatially to a non-membranous compartment. As a matter for future studies, the accumulation and mobility of proteins putatively inherent to the archaeal cell or genome architecture may result in nucleolus-like multifunctional complexes as subcellular organizers. For instance, if the putative nucleolus is multifunctional (e.g., eukaryotic nucleoli are involved in programmed cell death, metabolic regulation, cell differentiation, stress, and aging), then this may explain the presence of the many archaeal AgNOR-sensitive proteins that we identified that have no apparent functional relation to a eukaryotic nucleolus. The fact that we identified all these elements together in an archaeal nucleolus-like domain through the combination of microscopic and molecular approaches provides an initial understanding for a putative nucleoli in relation to archaeal cell organization. It also opens the door to address outstanding questions, such as how widespread the here-described nucleolus-like structure is across other groups of Archaea or Bacteria, or whether nuclear bodies (e.g., nucleolus, Cajal bodies, etc.) evolutionary predate the presence of a nuclear envelope.

Taken together, we think that our findings may be the beginning of a possible paradigm shift regarding the evolutionary origin of the nucleolus, the nucleus, and the cellular organization and complexity in the prokaryote-eukaryote divide. We posit that the first nucleoli have been discrete fibro-granular and argyrophilic domains whose nature may have been proteinaceous, gene-expression based, subcellular organizers, combining space, structure, and function in the erstwhile randomly distributed prokaryotic cytoplasm.

In support of this, a recent approach using chromosome capture (Takemata et al., 2019; Takemata and Bell, 2021) shows that Crenarchaeotes display a refined mechanism for chromosomal organization by coalescin proteins mediated grouping of distant loci, depending on levels of gene expression. Although we did not identify coalescin in our analysis, a putative archaeal nucleolus relates also to a form of subcellular organizer and chromatin organization. While coalescin is enriched in the so-called B chromosomal compartments that harbor fewer active genes, transcriptionally active A compartments containing rDNA loci are presumably particularly suitable for a nucleolus associated gene expression, organization, and regulation. Analogous to the concept of the eukaryotic nuclear architecture (i.e., Cajal and Polycomb bodies and puffs), gene expression in Archaea should relate to subcellular structures (like nuclear bodies) arranging genes, RNAs, and proteins, even in the absence of a nuclear envelope. Although past studies (Gaal et al., 2016) claimed that transcriptional active regions in bacteria were a reminiscence of the nucleolus, we think that the search for putative nucleoli should start in Archaea with an integrated approach. As outlined elsewhere (Takemata and Bell, 2021), guiding exploration based on phylogenetics and integrating microscopy, particularly TEM and super-resolution, to genomic, proteomic or transcriptomic approaches, e.g., 3C-Seq mapping, has the potential to provide prolific insights to the cell biology and evolution of nucleolus and ribosome biogenesis from Archaea to Eukarya. The same authors also suggest that a putative nucleolus may be possible in *S. solfataricus*, from a genome architectural point of view.

Despite our diverse lines of evidence, we want to emphasize that many technical limitations are still to be faced. For instance, it remains to be determined whether the here-observed AgNOR-stained subcellular structure(s) colocalize with rDNA/rRNA, due to the cytochemical nature of AgNOR stain that is not compatible to perform colocalization with UISH or antibodies in the same sample. Further, the use of transcription inhibitors such as actinomycin D, could be a greatly complementary approach to assess nucleologenesis, i.e., formation and disruption of nucleoli as inferred from the loss of AgNOR signal. We discern that such functional experiments, in addition to other approaches, such as incorporation of bromouridine to confirm transcriptional activity or the use of specific antibodies and RNA probes to localize proteins and snoRNAs *in situ*, will become an exciting perspective for the evolutionary cell biology community. Of note, actinomycin D reported doses are specific for eukaryotic polymerase I (Sirri et al., 2000), and inhibitors, antibodies, and *in situ* probes are still limited in Archaea research. Particularly, the development of a set of specific antibodies against archaeal ribosomal and nucleolar homologous proteins would be necessary to colocalize each protein.

On the contemporary picture of a two-domain tree of life, Eukaryotes are a branch within the TACK Archaea (Gribaldo et al., 2010). The presence of proto-nucleoli in species of the TACK Archaea suggests that the origin and evolution of the nucleolus traces back through archaeal phylogeny to diverse common ancestors, initially that of Eukarya and TACK-Archaea. It does not escape our minds that the presence or absence of this kind of proteinaceous organelles should be determined in more representatives of Archaea or even Bacteria: a motivation for emergent evolutionary cell biology. By mapping the presence and absence of nucleolar elements (molecular and structural) on the archaeal and bacterial phylogeny, we might be able to propose or reject a gradualist scenario of nucleolar evolution and grasp a more profound understanding of nanoscopic cell architectures.

## Data availability statement

All data needed to evaluate the conclusions are present in the manuscript, the Supplementary material, and the References therein. Amino acid translated genes of the genome of *S. solfataricus* strain P1 (GenBank accession number NZ_LT549890.1) are available at https://ftp.ncbi.nlm.nih.gov/genomes/all/GCF/900/079/115/GCF_900079115.1_SSOP1/GCF_900079115.1_SSOP1_protein.faa.gz. All protein features were annotated against the UniProtKB/SwissProt database available at https://ftp.uniprot.org/pub/databases/uniprot/current_release/knowledgebase/complete/uniprot_sprot.fasta.gz. GO annotations were obtained based on UniProt IDs by parsing a GOA gene association file available at http://ftp.ebi.ac.uk/pub/databases/GO/goa/UNIPROT/.

## Author contributions

PI-M conceptualized this research under supervision of LJ-G and CV, was involved in all stages of the project, is the main author, and wrote the manuscript. AC contributed to experimental design in genomics and proteomics, supported PI-M in related laboratory procedures, analyzed results from proteomics and genomics, and wrote the manuscript. MM supported PI-M with standardization of *S. solfataricus* cultivation, in Danielle Daffonchio’s laboratory at KAUST. LJ-G holds senior authorship, participated intellectually in the conceptualization and experimental design of this research as doctoral supervisor of PI-M, particularly supported PI-M on the UISH and TEM observations, and wrote the manuscript. The consolidated line of research of LJ-G is the structure, function, and evolution of the nucleolus. CV supported this project financially, delivered infrastructure for its completion, was intellectually involved in all stages of the project, and wrote the manuscript. All authors contributed to the article and approved the submitted version.

## Conflict of interest

The authors declare that the research was conducted in the absence of any commercial or financial relationships that could be construed as a potential conflict of interest.

## Publisher’s note

All claims expressed in this article are solely those of the authors and do not necessarily represent those of their affiliated organizations, or those of the publisher, the editors and the reviewers. Any product that may be evaluated in this article, or claim that may be made by its manufacturer, is not guaranteed or endorsed by the publisher.

## Footnotes

^1^ https://www.dsmz.de/collection/catalogue/details/culture/DSM-1616

^2^ https://www.dsmz.de/microorganisms/medium/pdf/DSMZ_Medium88.pdf

^3^ https://www.dsmz.de/microorganisms/medium/pdf/DSMZ_Medium182.pdf
