# Supplementary material for: Ultrastructural and proteomic evidence for the presence of a putative nucleolus in an Archaeon
Source: Front Microbiol. 2023 Feb 2;14:1075071. doi: 10.3389/fmicb.2023.1075071 (PMC9932318; doi:10.3389/fmicb.2023.1075071)
Supplement: Supplementary file 1 [file Data_Sheet_1.PDF]

Supplementary Figures

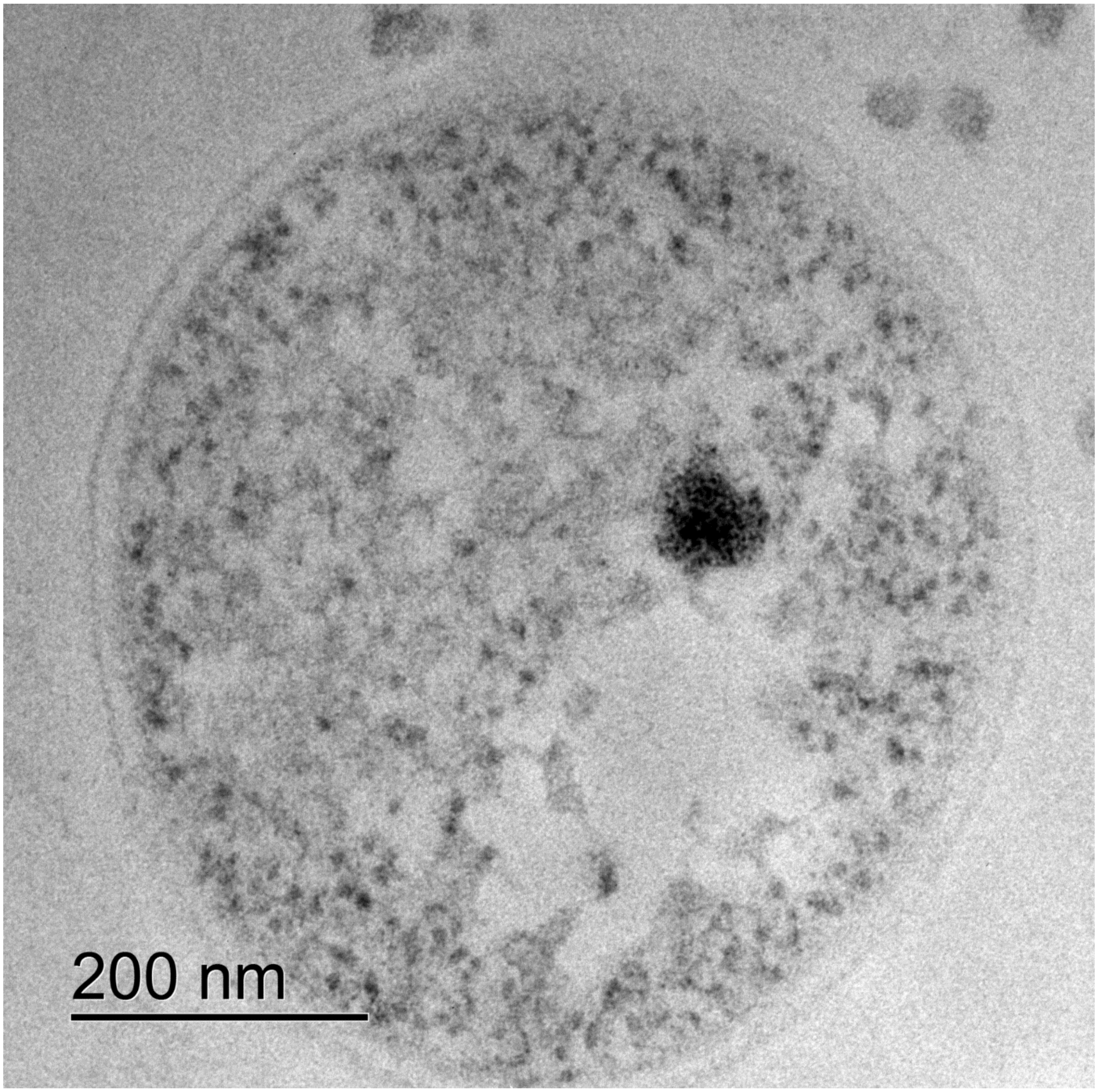

Figure S1. Single *Saccharolobus solfataricus (formerly known as Sulfolobus solfataricus)* cell with a nucleolus-like domain in which a fibro-granular morphology is evident.

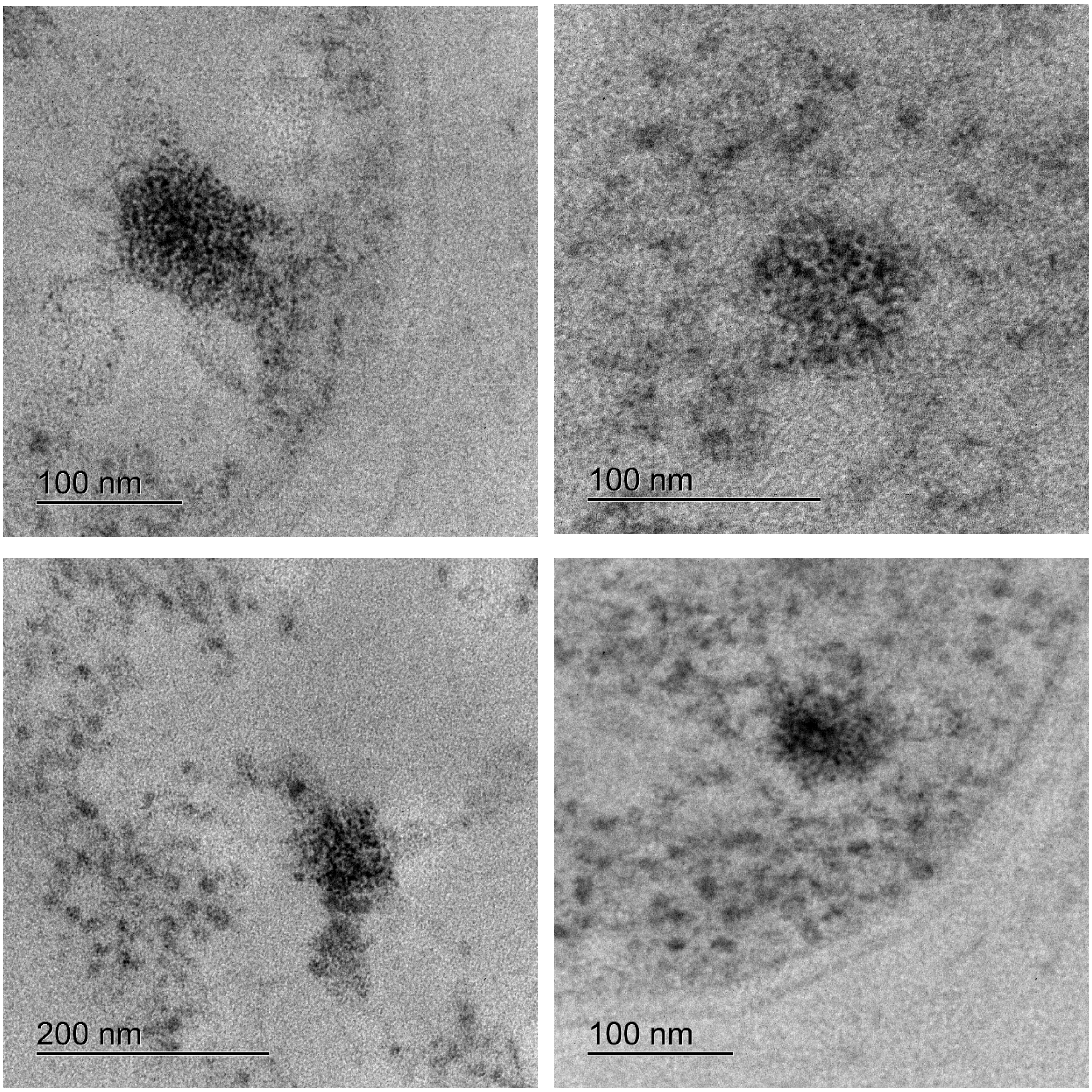

Figure S2. High magnification of four different nucleolus-like domains from four distinct cells of *S. solfataricus*. Granules and fibers are condensed within the respective nucleolus-like domains, contrasting in size and form with the surrounding cytoplasm. All four domains from four distinct cells share a common nucleolar morphology

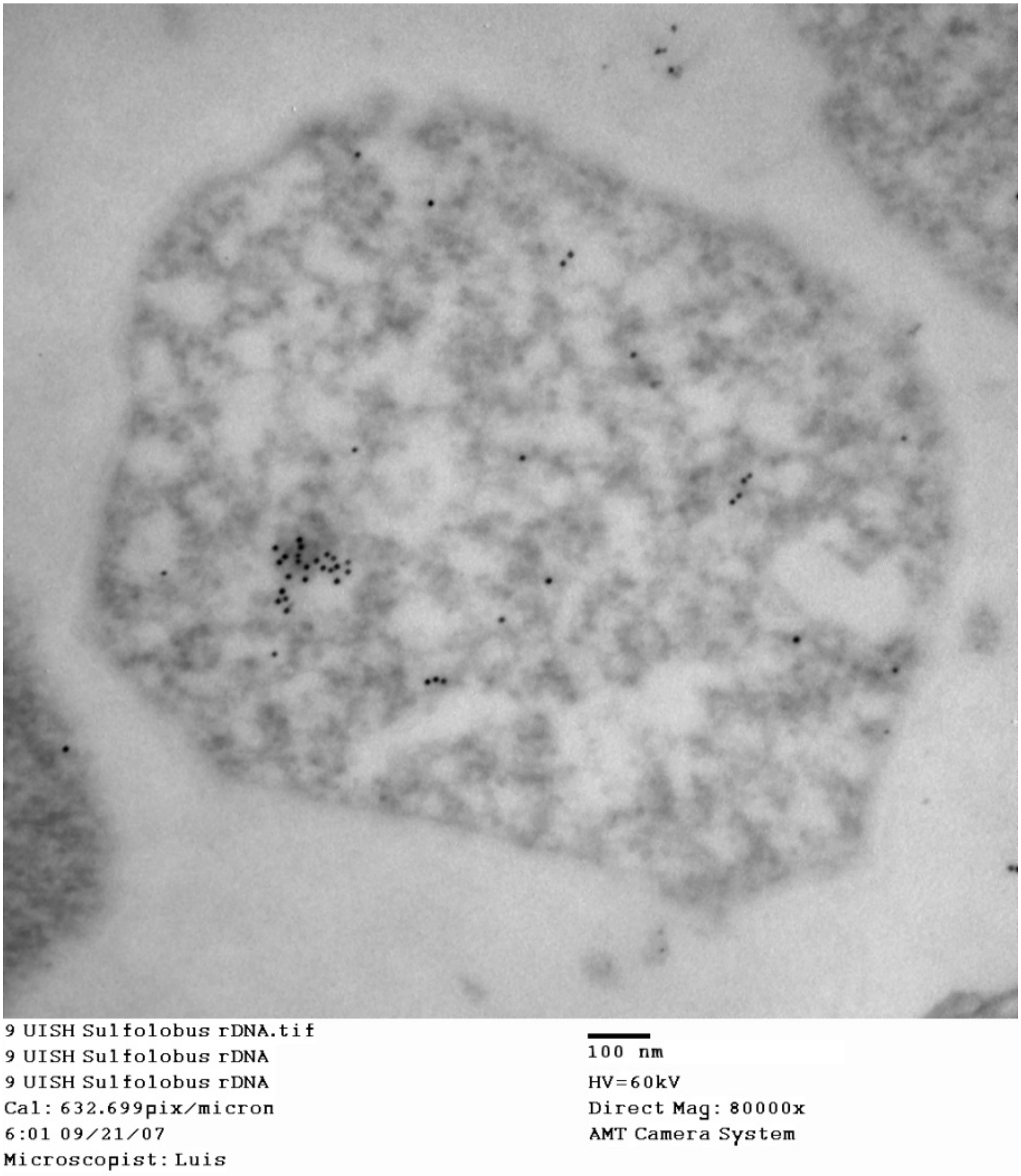

Figure S3. UISH of rRNAs under non-denaturalizing conditions.

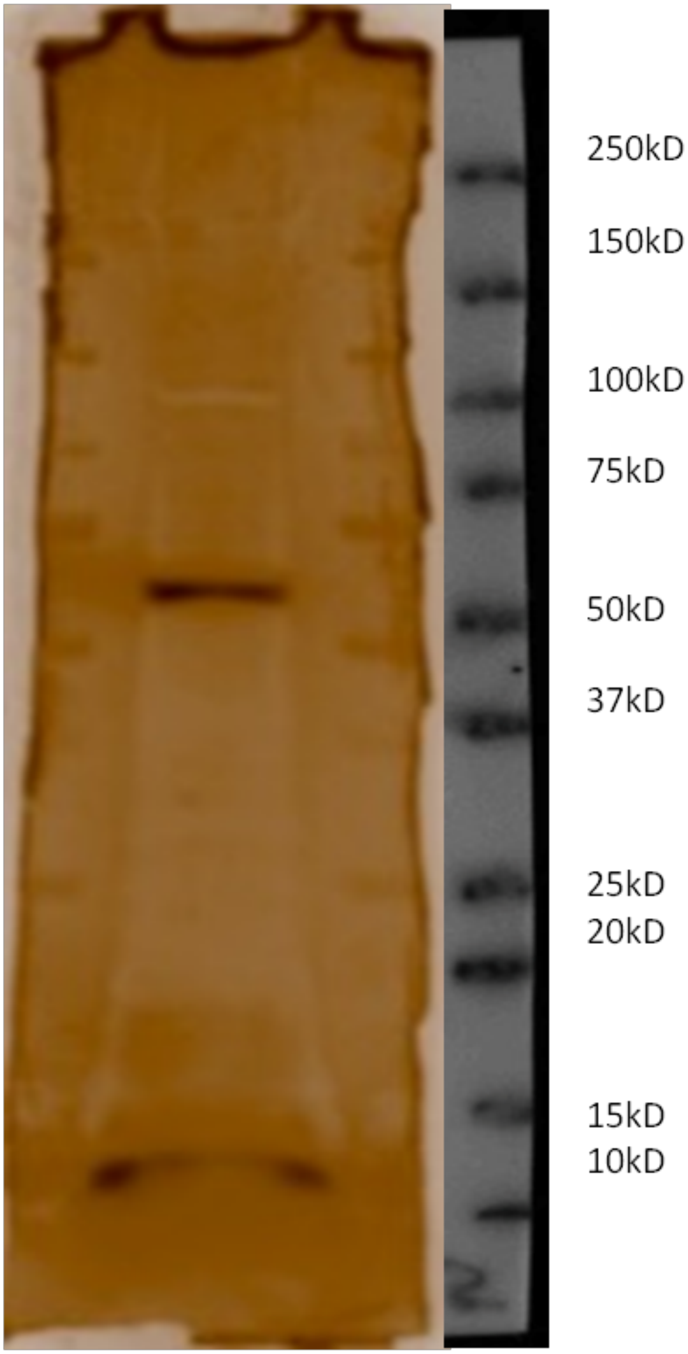

Figure S4. AgNOR banding pattern on SDS-PAGE form *Saccharolobus solfataricus* protein extracts. Black bands show concentration of argyrophilic peptides that were excised and sequenced with ancillary mass spectrometry. To the left is a peptide size reference marker.

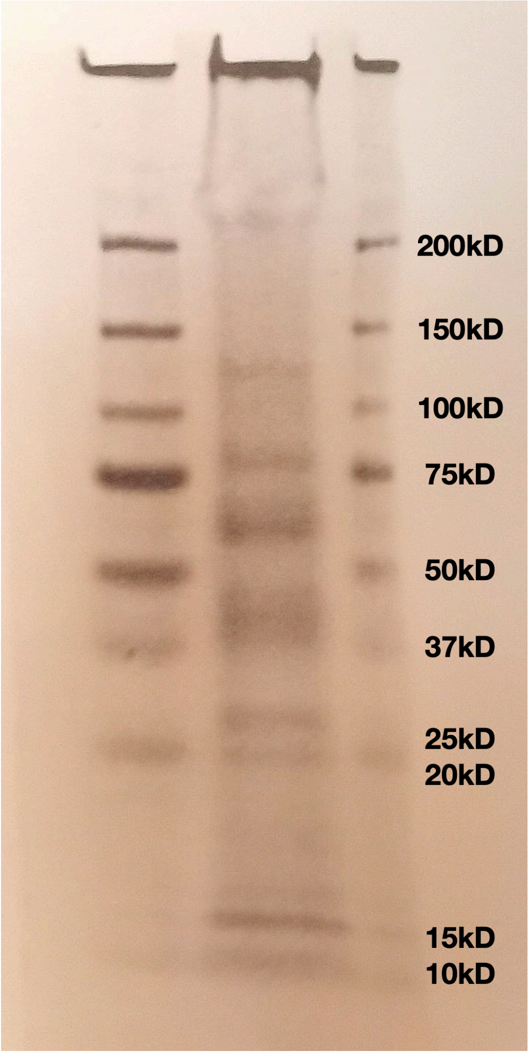

Figure S5. CBB stained banding pattern on SDS-PAGE from *Saccharolobus solfataricus* protein extracts. A diversity of bands is visible in comparison to staining with AgNOR. On both sides is a size reference marker.

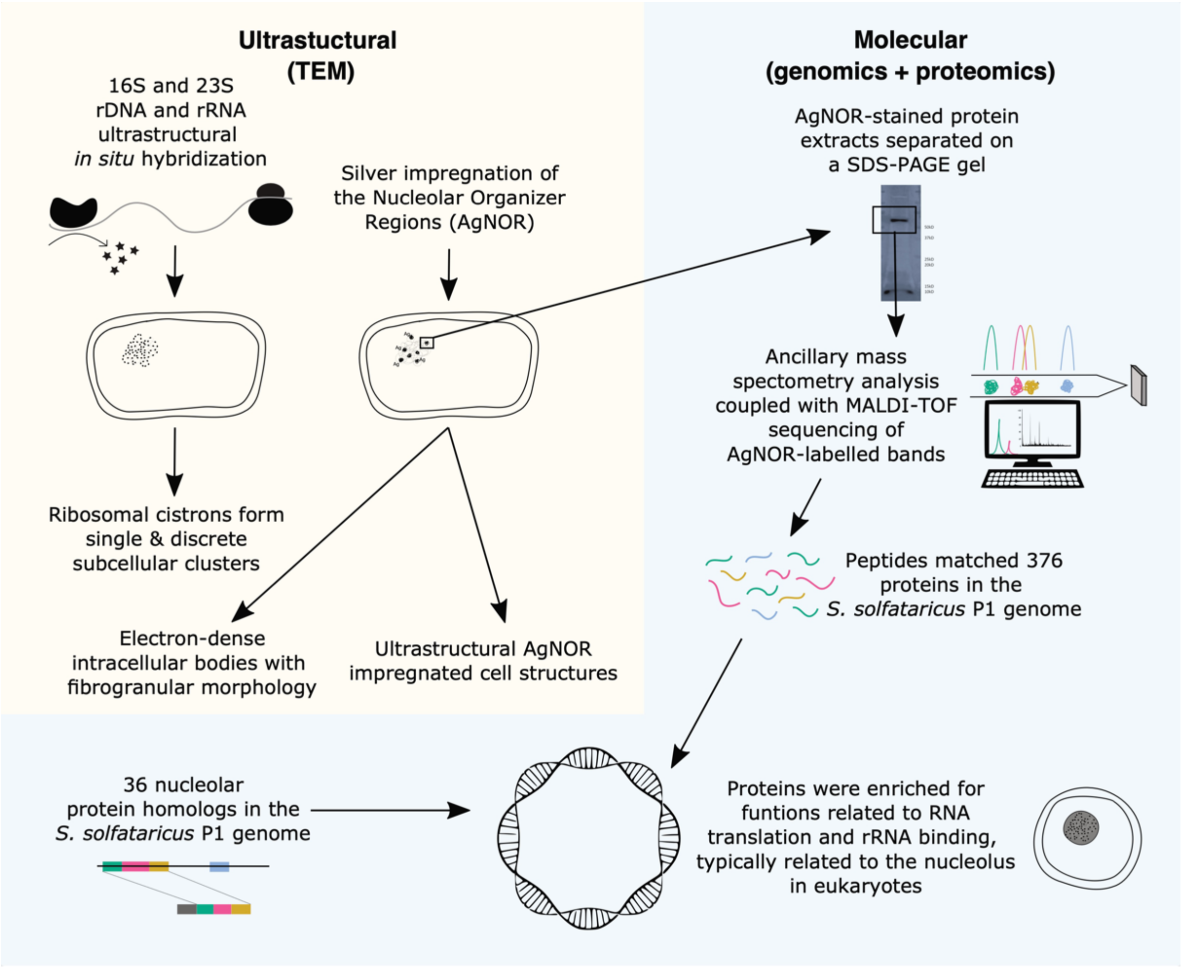

Figure S6. Methodology for Integration of ultrastructural and molecular approaches to assess the presence of a putative nucleolus in an Archaeon. 16S/23S ultrastructural hybridization and AgNOR staining are *in situ* techniques in TEM used to evidence nucleolus-like compartments. In *S. solfataricus* this is characterized by discrete clustering of ribosomal cistrons, fibro-granular morphology, and AgNOR positive discrete subcellular domains. *In vitro* SDS-PAGE analysis of AgNOR proteins with ancillary mass spectroscopy revealed 376 protein matches in the *S. solfataricus* genome, corroborated by the presence of 36 nucleolar protein homologs based on a candidate screen that putatively constitute within the newly described subcellular domain observed by TEM.

Supplementary Tables

Table S1. Precipitated proteins that aligned to translated genes of the *Saccharolobus solfataricus* genome. Local alignment was done using BLASTP (only 100% identical matches were allowed).

Table S2. Annotation of genes aligned to precipitated proteins.

Table S3. Gene Ontology enrichment of genes aligned to precipitated proteins from SDS-PAGE.

Table S4. Genes annotated to KEGG map ko03008 "Ribosome biogenesis in eukaryotes". Entries in bold denote the proteomic evidence by AgNOR staining.

Table S5. Nucleolus-related domains found in the *S. solfataricus* genome. Entries in bold denote proteins evidenced by AgNOR staining and MS peptide sequencing.

Table S6. Summary of nucleolus related protein domains found in *S. solfataricus* and their distribution across the tree of life. Entries in bold denote the proteomic evidence by AgNOR staining.
